# A Novel Information Retrieval Tool to Find Hospital Care Team Members: Development and Usability Study

**DOI:** 10.2196/humanfactors.6781

**Published:** 2018-04-16

**Authors:** Kyle Morawski, Craig Monsen, Sukhjit Takhar, Adam Landman

**Affiliations:** ^1^ Atrius Health Boston, MA United States; ^2^ Emergency Medicine Brigham & Women's Hospital Boston, MA United States

**Keywords:** communication, patient care team

## Abstract

**Background:**

Hospital communication among members of a patient’s care team is a central part of clinical workflow and consumes a large amount of a health care provider’s time. Oftentimes the complexity of hospital care leads to difficulty in finding the appropriate contact, which can lead to inefficiencies and frustration. Squire is a Web-based information retrieval app created to improve the speed and efficiency in reaching the appropriate team member during the care of a hospitalized patient.

**Objective:**

The objective of the study was to design and develop Squire and to evaluate the usage, usability, and perceived effect of the app on finding the correct contact within a hospital.

**Methods:**

We used a mixed-methods design using a before-after survey methodology combined with one-on-one interviews to understand the perceived effect of Squire. The study took place at an academic medical center with internal medicine resident physicians. We surveyed residents on demographics, as well as time and efficiency of hospital communication before and after the use of Squire. After using Squire, participants were also asked to evaluate Squire’s Net Promoter Score (NPS). A subset of voluntary participants participated in one-on-one interviews and completed the System Usability Scale (SUS). We performed descriptive statistics on participant characteristics, app usage data, and responses to surveys. Survey results were compared before and after Squire adoption using the Wilcoxon rank-sum test and a general linear model. Interview data were analyzed using content analysis with a qualitative description approach to review and categorize feedback from participants.

**Results:**

There was a 67.9% (74/109) response rate to the pre-Squire survey and 89.9% (98/109) response rate to the post-Squire survey. At baseline, there was an average of 22.2 (95% CI 18.4-26.0) minutes/day spent searching for the right contact, and this decreased to 16.3 (95% CI 13.9-18.7) minutes/day after Squire was launched (*P*=.01). There were favorable usability scores, with an average SUS of 84.7, and a marginal NPS of +6.1. Overall, the use of Squire included 22,283 page views, most commonly to contact the admissions office or portable chest x-ray technician. Interviews highlighted common benefits of Squire, including decreased perceived time spent on hold with operators and improvement in connecting with the appropriate contact in specialized, complex departments. Future opportunities were also identified to improve Squire including adding a two-way communication between physician and nursing staff and providing offline access.

**Conclusions:**

Squire decreased the perceived time required to find an appropriate contact and had a favorable usability score; however, the NPS was marginal and several opportunities were identified to improve Squire for future use.

## Introduction

### Background

The complexity of current medical care requires frequent communication within the care team, but many systems do not allow this to be done efficiently. Most academic medical centers have grown piece-by-piece, rather than being designed to function as a coherent whole. In fact, communication has become so centralized that some hospitals are devoting entire departments to this endeavor [1]. Previous studies have shown that the amount of time spent talking to providers is almost double than that of direct patient care [2]. Patient safety has also been shown to be dependent on good team communication [3], and the economic burden of communication inefficiency has been estimated at US $12 billion per year in the United States [4].

At our hospital, there are 2 main workflows for contacting the most appropriate care team member: (1) one can call the hospital operator and wait to be connected or (2) utilize the hospital’s Web-based paging directory and search for the correct contact. Many people find wait times with the operator long and the paging directory difficult to search. Both can be ineffective because of poor matching and unclear role description. While the immediate care team members (attending physician, resident physician, nurse) are listed in the electronic health record (EHR), other care team members such as the respiratory therapist, echocardiogram technician, or radiologist can be more difficult to locate.

### Importance

Up to one-fifth of a medical intern’s time is used for talking with other providers, representing the single largest activity performed during a workday [2]. In a 2013 study from Johns Hopkins, talking with other providers was more time-consuming than direct patient care, which represented 12.3% of a medical intern’s time. As this is a large portion of one’s time and care becomes more complex, it will be necessary to optimize how we identify and contact members of a patient’s health care team.

Technology has been lauded as a solution to help improve health care delivery efficiency. If the benefits of technology are to be realized, such that the health care system is able to achieve improved value in the setting of expanding complexity of patients and care, there must be a focus on the human factor in care redesign and process flow [3,5]. There can often be unintended consequences of introducing new technologies, therefore evaluating users’ response to a new tool is important to ensure that desired positive impacts are achieved [6].

### Goals of This Intervention

We designed and implemented a novel Web-based information retrieval app, Squire, to improve the speed and efficiency to reach the appropriate team member during the care of a hospitalized patient at a large academic medical center. With increasing complexity of care, work hour restrictions, and demands for productivity in current hospital medicine, Squire aims to facilitate contacting the correct member of a patient’s care team and to reduce the need to call the hospital operator. All interface construction, back-end programming, and user experience was focused on speed of activity completion. In this paper, we describe the design and development of Squire and then evaluate the usage and usability of the platform, as well as its perceived effect on efficiency in finding the correct contact in a real-world setting.

## Methods

### Study Design and Setting

In this mixed-methods study, we evaluated the Squire app using a before-after survey methodology combined with purposefully selected, semistructured individual interviews. We performed the study in an academic medical center, with internal medicine resident physicians using the app during their usual clinical practice.

The Partners Health Care institutional review board (Partners Health Care, Boston, MA) deemed this study exempt from review.

### Intervention: Squire

**Figure 1:**
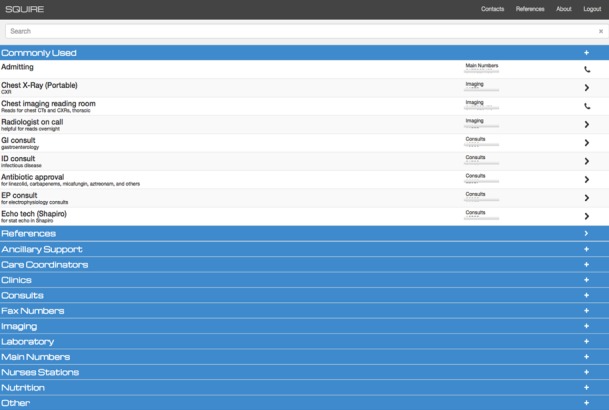
The Squire application landing page with most commonly used contacts displayed.

Squire is a Web- and mobile-based software app designed to offer clinicians with quick access to commonly used resources, including hospital back office phone numbers, the hospital paging system, and clinical references (Figure 1). Squire was conceived and developed by one of the authors (CM), a dually trained internal medicine physician and clinical informaticist, using an iterative, user-centered design approach [7]. Given CM’s expertise with the app context, requirements, and capabilities, he created the initial concept and design. A small cohort of pilot users provided critical feedback including the most useful contact numbers, broken links, and appropriate groupings of contact information. In these early feedback sessions, as well as in previous research [8,9], it was clear that speed and simplicity were of paramount importance. These users also provided iterative feedback on mock-ups and prototypes, leading to interface improvements to optimize usability and satisfy real-world settings [7].

Users log in via computer workstations or mobile phones through the internet, using their hospital clinical system credentials. Phone and pager numbers are listed in a searchable directory. As distinct from existing tools, the directory includes indexed, searchable comments that may be modified based on user feedback in addition to titles, phone numbers, and pager numbers to aid in identifying the most appropriate contact. These contacts include consult services, radiology reading rooms, laboratory departments, nurses stations, pharmacists, care coordinators, and nearby hospitals among others. Users may initiate a call directly from their phone by selecting the contact or sending a text page by selecting a pager contact and entering a message into a structured paging Web form.

The app is delivered as a website hosted on an internal Partners Health Care CentOS Linux server. The site is accessible on all platforms including mobile using responsive design JavaScript, CSS (cascading style sheets), and HTML5. Responsive design means that the appearance of the website adjusts dynamically to where and how the user is viewing it, responding to features such as the device, browser, and window size. This technique is now ubiquitous for highly trafficked websites. Bootstrap and JQuery, open source frameworks, were used for the front-end user interface using a moderate amount of custom JavaScript and CSS to optimize the experience.

**Figure 2:**
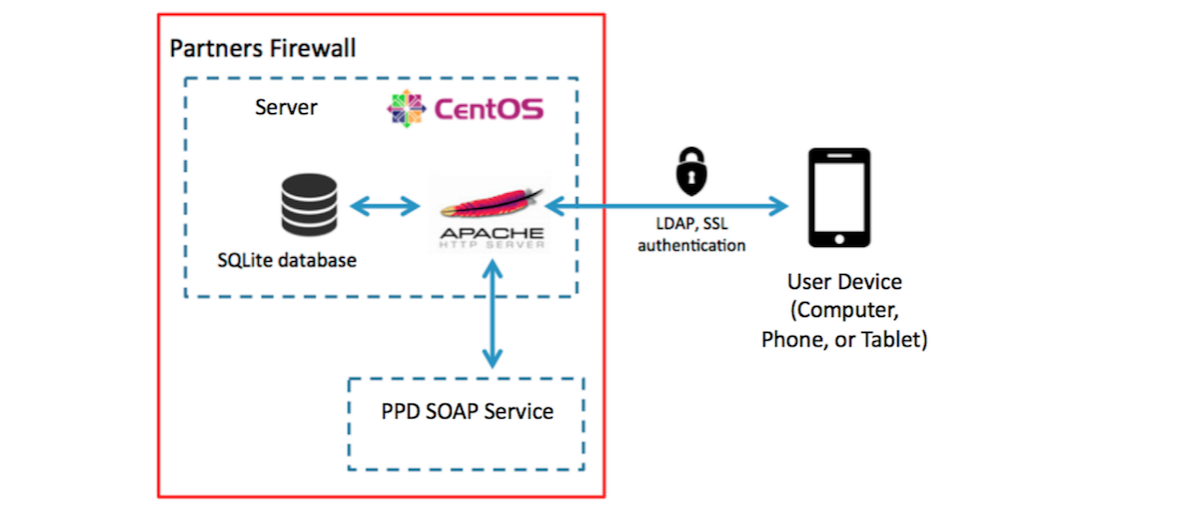
The Squire technical architecture. LDAP: lightweight director access protocol; PPD: partners phone directory; SOAP: simple object access protocol; SSL: security service provider.

The back end architecture also consists of open source technologies, including Ruby on Rails served using an Apache HTTP server. Data are stored on a SQLite database. It should be noted that there is no personally identifiable or protected health information stored on the server or in the app. Figure 2 summarizes the system’s technical architecture.

There are two noteworthy integration points for the software: (1) user authentication and (2) paging. First, we integrate with the hospital’s lightweight directory access protocol (LDAP) servers so users can use their hospital clinical systems credentials to log in to Squire. User authentication is performed by the server after a user has entered in their credentials via a secure socket-layer (SSL)-enabled, encrypted LDAP adaptor. This securely checks against the hospital’s LDAP servers so that a user with the provided username and password is authorized before allowing access to the app. Second, text pages can be sent directly from the Squire app as a result of integration with our hospital’s Paging Directory Service. This is a simple-object access protocol (SOAP)-enabled service through which “3rd party” apps can be built to send pages on the hospital’s paging network [10]. It also allows apps to search the directory to match users to pager numbers and to determine which users are currently accepting pages.

### Participant Selection

Internal medicine resident physicians at a large academic medical center were provided access to Squire between January 2015 and February 2016. We selected internal medicine residents because they frequently need to identify and communicate with other patient care team members, such as specialty physicians, care coordinators, and respiratory therapists. At this institution, there are 109 residents in internal medicine annually, of which 44% are female, with an average age of 30 (range 25-42) years.

### Study Protocol

**Figure 3:**
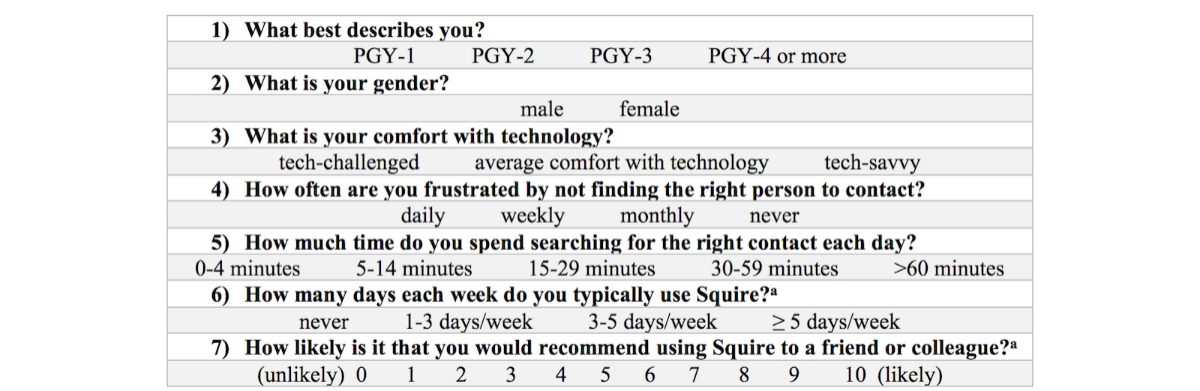
Evaluation survey pre- and post-Squire use. The letter a signifies only present on the post-implementation survey.

Before availability of Squire, we emailed the residency with a baseline survey (Figure 3) of their contact searching challenges, including how often they are frustrated by not finding the right person to contact and how much time they spend searching for right contacts each day.

Squire was made available to all internal medicine resident physicians in February 2015 by an announcement at a resident conference and sending email notifications. We allowed resident physicians to use Squire for 6 months and then sent an evaluative survey using Research Electronic Data Capture (REDCap, Vanderbilt University, Nashville, TN), a secure, Web-based app designed to support data capture for research studies [11]. The post-Squire survey mirrored the baseline survey, with the addition of Net Promoter Score (NPS) and a question regarding use of Squire in everyday practice (Figure 3, question 7). NPS [12,13] is used by many to evaluate how likely someone is to recommend the new product or technology to a friend or family member. Survey respondents were also asked whether they would be willing to participate in a follow-up one-on-one, semistructured interview about their use of Squire. Participants had the option to stop the survey at any time.

**Figure 4:**
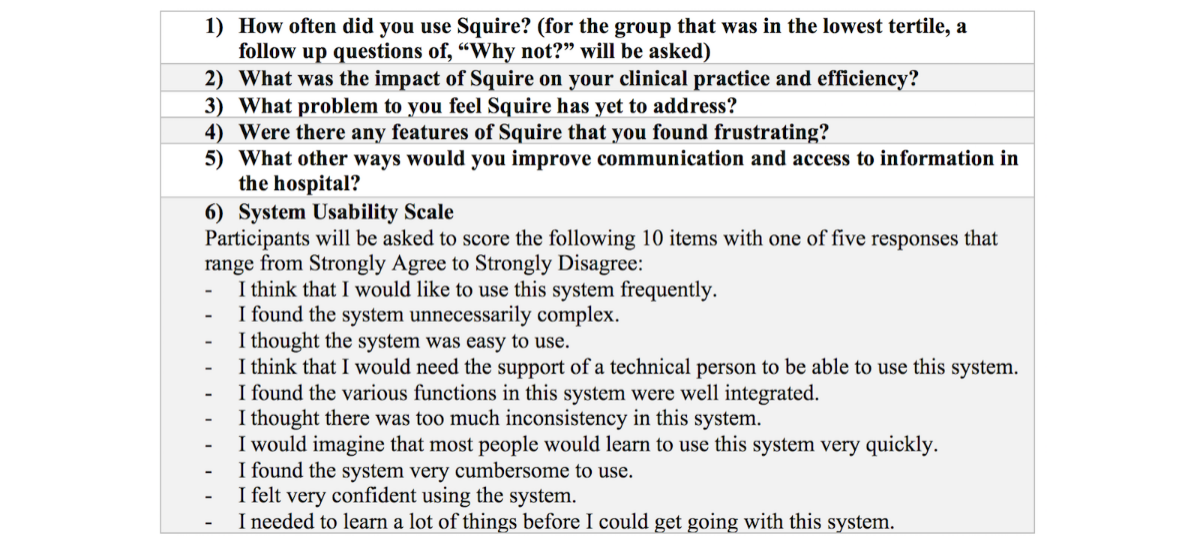
Semistructured interview guide.

There were 98 responses (90% response rate) to the survey, with 81 of the respondents (83%) indicating acceptance to be interviewed. Survey participants willing to be interviewed were arranged in tertiles with respect to number of log-ins to Squire, and 9 interviewees (10% of total respondents), were purposefully selected [14] from this list, blocking on number of log-ins. The interviewer used a one-on-one, semistructured approach [15] with an interview guide, but with allowance for the interviewee to bring up themes at their discretion (see Figure 4). The interview ended with the System Usability Scale (SUS; see Figure 4, question 6), a validated measure of system usability [16,17]. The interviewer took notes and audio-recorded the interviews. Participation in the one-on-one interviews was voluntary; participation and feedback provided did not impact professional standing or performance evaluations. All qualitative interview participants provided verbal informed consent and were compensated with a US $30 gift card for attending the interview process.

In addition to user experience evaluation described above, we tracked Squire usage statistics through audit logs, including number of log-ins, commonly used features, and total number of users over time.

### Outcome Measures

Overall, we evaluated Squire’s usage and usability, as well as the perceived effect of Squire on efficiency of finding the correct contact in the hospital setting. We measured Squire usage through logs of unique users for Squire, frequency of page views overall, and frequency of specific page views to identify most commonly used features. We also surveyed the users on how often they used Squire (Figure 3). Usability was measured by the SUS, NPS, and exploratory, qualitative semistructured interviews with the users. Time spent searching for the appropriate contact was measured before and after Squire implementation by a 5-category Likert scale survey question (Figure 3) with the following intervals: 0-4 min, 5-14 min, 15-29 min, 30-59 min, and >60 min.

### Analysis Approach

We present participant characteristics, overall use, and most commonly used features of the Squire app with descriptive statistics. To analyze the survey results before and after the use of Squire, we used the Wilcoxon rank-sum test. Since ordinal category differences can be difficult to interpret, we also performed an adjunct analysis to estimate the average time saved with the Squire platform, an approach supported by prior research [18]. We compared the mean time spent searching for the right contact each day before and after Squire implementation using a general linear model with a link function and robust variance to show magnitude of findings, using each ordinal unit’s midpoint [19,20]. For those who spent over 60 min, we used 70 min as the mean time spent searching for the right contact, providing a conservative estimate, minimizing the effect of outliers.

A content analysis [18,21] was performed on the one-on-one interview data using a qualitative description approach. Two of the investigators (KM and CM) reviewed notes and audio recordings, coding and sorting content to identify key phrases and meaningful text units. Both investigators performed this task independently, then met to discuss categories and subcategories of feedback, iteratively revising until consensus was reached. These investigators selected representative quotes for each of the categories identified, extracting quotes from the audio recordings to ensure accuracy.

Qualitative data were managed using Microsoft Excel (Microsoft Corporation, Redmond, WA); quantitative data were analyzed in STATA 14 (StataCorp, LLC, College Station, TX).

## Results

### Characteristics of Study Subjects

There was a 67.9% response rate (74/109) in the baseline survey, and an 89.9% response rate (98/109) in the follow-up survey. Characteristics were similar between the 2 groups with respect to postgraduate year, sex, and level of comfort with technology (Table 1).

### Survey Results

#### Survey Response

In the baseline survey, 97% (72/74) of respondents felt that they were frustrated by the difficulty in finding the right person to contact either daily or weekly (Table 2). None responded that they were never frustrated by inability to contact the right person. Nearly three-fourth of the respondents felt that they spent 30 min or less a day searching for the right contact, whereas the remainder felt that they spent more than 30 min daily.

**Figure 5:**
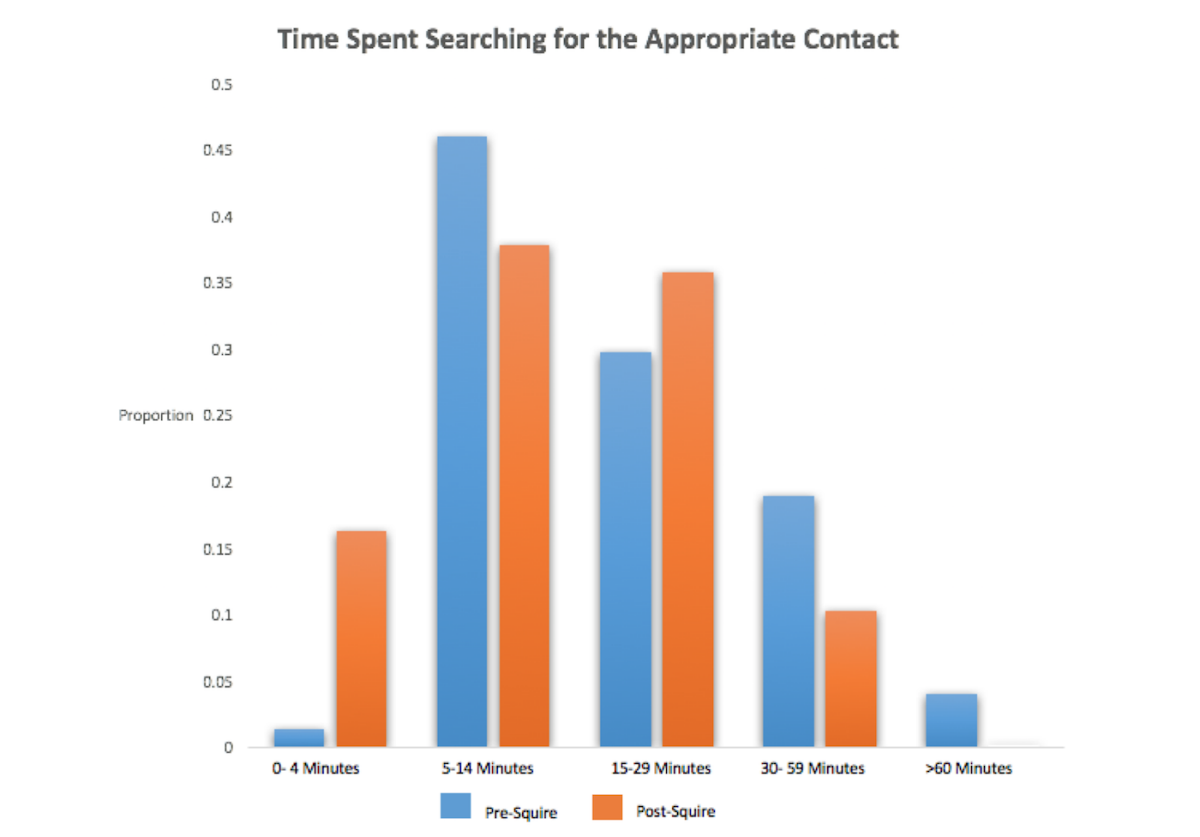
Time spent searching for appropriate contact pre- and post-Squire use.

After implementation of Squire, we observed a significant decrease (*P*=.02) in the amount of time spent in finding the right person to contact (Figure 5 and Table 2). In our regression model, we also found that participants spent 5.8 min (95% CI 1.6-10.2) less searching for the right contact each day after Squire implementation. There were still no participants who were never frustrated by trying to find the right person to contact.

**Table 1:** Characteristics of survey participants pre- and post-Squire. PGY: postgraduate year.

Characteristics of survey participants		Pre-Squire (n=74), n (%)	Post-Squire (n=98), n (%)	*P* value^a^
**Resident training level**				.28
	PGY-1	35 (47)	36 (37)	
	PGY-2	20 (27)	37 (38)	
	PGY-3	16 (22)	22 (22)	
	PGY-4 or more	2 (3)	3 (3)	
Female		42 (57)	38 (39)	.55
**Technology comfort level**				.15
	Tech-challenged	1 (1)	3 (3)	
	Average comfort	53 (72)	62 (63)	
	Tech-savvy	14 (19)	33 (34)	

^a^*P* value for group differences calculated with Wilcoxon rank-sum test.

**Table 2:** Comparison of care team communication efficiency pre- and post-Squire and reported use of Squire.

Care team communication		Pre-Squire, n (%)	Total minutes searching per day^a^	Post-Squire, n (%)	Total minutes searching per day^a^	*P* value
**How often were you frustrated by not finding the right person to contact?**						.66^b^
	Daily	40 (54)		58 (59)		
	Weekly	32 (43)		34 (35)		
	Monthly	2 (3)		6 (6)		
	Never	0 (0)		0 (0)		
**How much time do you spend searching for the right contact each day?**						.02^b^
	0-4 mins	1 (1)	2	16 (16)	32	
	5-14 mins	34 (46)	323	37 (38)	351	
	15-29 mins	22 (30)	484	35 (36)	770	
	30-59 mins	14 (19)	623	10 (10)	590	
	>60 mins	3 (4)	140	0 (0)	0	
Average time searching for contact per person per day (95% CI)			22.2 (18.4-26.0)		16.3 (13.9-18.7)	.01^c^
**How many days each week do you use SQUIRE?**						
	Never			34 (35)		
	1-3 days/week			46 (47)		
	3-5 days/week			8 (8)		
	>5 days/week			10 (10)		

^a^Midpoint from range of time multiplied by n (ie, midpoint of 5-14 mins is 9.5 mins, multiplied by 34 participants who selected that range, results in a total of 323 minutes searching per day).

^b^*P* value for group differences, calculated with Wilcoxon rank-sum test.

^c^*P* value calculated using general linear model with robust variances.

A majority (74%, 72/98) reported spending between 5 and 30 min a day searching for the right contact; however, 16% (16/98) reported spending less than 5 min a day searching, equating to an absolute increase of 15% with respect to pre-Squire survey.

Use of Squire was reported as being typically less than 3 times each week by 82% (80/98) of respondents; however, there was a small proportion (10 respondents, 10%) who used it 5 or more times each week.

#### Net Promoter Score, System Usability Scale

Of the 98 respondents to the postimplementation survey, 32% (32/98) scored the likelihood of recommending Squire to a friend or colleague as 6 or below on a 10 point Likert scale, and thus were classified as detractors. Thirty-nine percent (38/98) of the respondents scored this same question as a 9 or higher and were classified as promoters, and 20% (20/98) respondents provided a score of 7 or 8 and were classified as neutral. This provided an overall NPS of 6.1. The SUS resulted in a mean score of 84.7 on a scale of 0 to 100. The scores ranged from 70 to 97.5.

#### Most Commonly Used Features

During the 6-month period between launching Squire and performing the evaluation, there were 312 unique users and 22,283 page views. The most commonly viewed features were to contact the admission office (279 views, 2.3% of total views), portable chest x-ray technician (240 views, 1.1% of total views), or the chest imaging reading room (234 views, 1.1% of total views).

### Qualitative Interview Results

Participants identified 3 major categories of feedback on Squire during the one-on-one interviews: (1) reducing hold time with hospital operators; (2) value in complex, specialized departments; and (3) opportunities for improvement. Table 3 summarizes these categories and provides additional illustrative participant quotes.

#### Use of Squire Reduces Time Spent on Hold With Hospital Operators

Seven respondents commented that the largest impact on efficiency in the hospital is with not having to wait on hold with the operator while being transferred to the desired contact. It was cited that this could save, “5 minutes with each call,” and, “has allowed…patients to get more timely care” (Participant 9, Post Graduate Year (PGY) 2). Furthermore, 3 respondents indicated that finding the appropriate number to call was only in Squire and not present with the current Web-based paging directory. Participant 1 (PGY 2) explained, “There are so many headaches during residency, and trying to find the right number shouldn’t be one of them” (see Table 3).

**Table 3:** Key categories and themes identified during one-on-one interviews with illustrative quotes from participants. PGY: postgraduate year.

Theme		Example quote(s)
Use of Squire reduces time spent on hold with hospital operators		“Could quantify the time it could peel off the day or week.” [Participant 1, PGY-2]
		“It just makes everything quicker, I used to wait on hold with the operator, now I can just look it up.” [Participant 8, PGY-1]
Squire is particularly valuable for finding contacts from specialized, complex departments		“There are so many headaches during residency, and trying to find the right number shouldn’t be one of them.” [Participant 1, PGY-2]
		“Could save me 5 minutes depending on how many wrong phone calls I make or get connected to the wrong places.” [Participant 9, PGY-2]
**Opportunities for improvement of Squire**		
	Two-way communication with the nursing staff	“There is a lot of ‘cat and mouse’ with trying to call back [nursing staff], especially on night float.” [Participant 3, PGY-1]
		“If in Squire we knew the nurse’s name and contact information it would speed things up.” [Participant 9, PGY-2]
	Offline access	“If you could just take out your smartphone and use the features without waiting for a connection to login that would be great.” [Participant 4, PGY-3]

#### Value for Finding Contacts From Specialized, Complex Departments

Nearly all of participants who were interviewed indicated Squire helped most with finding the appropriate person to call from specialized and complex departments. Two areas that were referenced multiple times were the radiology department and the care-coordination department. In these circumstances, the extra numbers provided in Squire were felt to increase efficiency by requiring less inappropriate calls and redirection to the correct contact. One PGY 2 participant commented that, “It’s almost as if [specialty service] wants paging to be frustrating.”

#### Opportunities for Improvement: Two-Way Communication and Offline Mode

There were several areas reported as needing further work. Four interview participants indicated that two-way communication with nursing would be necessary to improve communication efficiency and decrease hold times. They all described instances of being paged by a nurse to the central nursing station and having to wait while the nurse who paged them was found. Many participants also indicated that the need to log in was a barrier to use of Squire. Recommendations for enabling the app function offline (without live network connection) with incremental updates as needed were suggested to improve the usability and efficiency.

## Discussion

### Principal Findings

We described the design and development of a Web-based information retrieval app, Squire, to improve the speed and efficiency of finding the appropriate contact of a hospital care team. In a pilot with internal medicine resident physicians, 301 users accessed 22,000 page views; however, the majority of users reported only limited use each week. Users reported a strong SUS of 84.6 but a marginal NPS of 6.1. In qualitative interviews, participants provided constructive feedback on features that could be improved. We found that users spent 5.8 min less self-reported time searching for contacts per day after Squire implementation, although there was no change in user frustration levels. While a savings of 5.8 min per day may seem small, when averaged over longer time periods and a population of clinical users, the time savings is substantial.

The most commonly used features were to call the admissions office and the radiology technicians. In general, these are commonly accessed hospital departments but may represent a gap in our institution’s current paging directory that does not easily provide these frequently used numbers. These two department numbers are also visible on the front page of the Squire app without any additional searching or scrolling. The qualitative interviews found that Squire was particularly valuable for specialized, complex departments. Radiology is an example of a complex department, with multiple imaging modalities (technicians) and specialty radiology reading rooms (radiologists) spread across a large campus. This inherent complexity and large number of radiology phone number options may also explain why radiology was a commonly used Squire feature.

We expected that Squire would be a frequently used information retrieval tool; however, we found that approximately half of the survey respondents reported that they used Squire 1 to 3 days/week, a larger than expected percentage of respondents (35%) reported that they never used Squire, and only 18% of respondents reported using Squire for 3 or more days/week. These seemingly low reported usage patterns suggest a limit to the value of Squire in everyday clinical practice. Some users may be reserving Squire use for cases in which the phone numbers are difficult to locate via other methods. We also noted that resident physicians rotate roles and call schedules and therefore may have variable need for Squire in any given week. Since we did not specifically ask users to explain their usage frequency, further research is needed to understand Squire usage patterns and whether this reflects limitations of Squire functionality and usefulness.

Squire received a favorable SUS of 84.6, well above the generally accepted average SUS score of 68 [17,22], indicating that Squire was intuitive and easy to learn. Previous research indicates that a score above 82 corresponds to someone being a “promoter” of a new technology [17]. In contrast, Squire’s NPS of 6.1 was marginal. Overall, an NPS greater than zero is “good,” as positive scores mean that there are more promoters of the product than detractors. Thresholds of 50 have been described as “excellent” and above 70 as “world class.” [23]. The Temkin Group benchmarks NPS by industry sectors and found that software had a mean NPS of 41 with a range of 28 to 55 [24]. If we benchmark against health care software, 4 Acute EHRs had NPS of −65, −64, −38, and 0. On balance, Squire’s NPS of 6.1 is outstanding compared with EHRs but mediocre when compared with other software companies suggesting an opportunity to improve Squire and guide further iterations over time with serial internal NPS measurements [12].

While Squire’s NPS was marginal, we also observed actual promotions of the product to additional users. When Squire was deployed there was no incentive to use it or recommend it to others, yet after 6 months, there were 312 unique users; however, Squire was only rolled out to the 109 internal medicine residents as a part of this study. The observation that app use has naturally diffused outside of the initial study group suggests that there is value to the app outside of the studied individuals and provides some support that positive findings would generalize to clinicians outside of the study population.

A priori, we expected time to search for contacts and user frustration to be correlated. We found that time to search was reduced after the introduction of Squire but frustration levels were not significantly different statistically. Given our small sample size, it is possible that we did not detect a small change in frustration. Furthermore, it is possible that larger time savings are needed to change frustration levels and that we may not have reached these levels with Squire. Qualitative interview feedback confirmed that Squire helped reduce the time that physicians spent waiting on hold for the operator or calling the incorrect contact, but there may also be other factors impacting frustration, such as hold time and redirection to another contact even when the correct phone number is called. Further work is needed here, as efforts to improve the efficiency of nonpatient care activities, have the potential to increase focus for physicians on more critical patient care activities, reduce frustration, and improve the overall efficiency of health care delivery.

### Comparison With Prior Work

Entrepreneurial endeavors exist to create a mobile phone app to simplify phone directories [25] or improve access to clinical references [26]; however, research on usability or impact on clinical practice is lacking. There exists previous research regarding development and usability of physician directories [27]; however, effectiveness of implementation remains a poorly studied topic.

Squire was developed to improve efficiency in finding the correct contact among the care team of a hospitalized patient. Mobile usage in the hospital has been increasing, with the main reason cited being speed [28]. In order to integrate mobile devices and new technology into incumbent processes, they must be seamless and represent minimal practice change [13]. Squire attempted to address these issues through working with an already present paging and directory system, allowing for clear descriptions of appropriate numbers to call, and integrating into a mobile interface so that paging and calling can occur directly from a mobile device. Further development is still required in this arena, with this study suggesting that two-way communication would be welcomed by users. This sentiment has been described previously [6] and been shown to improve closed-loop communication [29].

### Limitations

There are several limitations to this study that are inherent to its design. First, the study was performed at a single institution with a single group of internal medicine residents, so the generalizability to other institutions and specialties may be limited; however, we had excellent response rates among those requested to participate, which adds to the validity of responses and representativeness of the results to our institution’s internal medicine residents. While there were not significant differences between the initial and post-Squire survey participants, there was a slight increase in PGY-2 representation (27% initial and 38% post-Squire). This increase in more experienced survey respondents could contribute to improvements in time to find the correct contact.

We did not capture participant identifiers for the baseline or post-Squire surveys, and therefore it is unknown up to what extent baseline survey respondents are also represented in the post-Squire survey. Furthermore, we were not able to account for the repeated measures in the statistical analysis.

We used a small sample size for the qualitative interviews. Our content analysis approach identified key, common themes; however, it is possible that additional concepts or themes would have emerged with additional participants.

The launch of Squire coincided with the implementation of a new EHR system in our hospital, which may have impacted residents’ self-assessment of efficiency. Previous research supports that physicians are more likely to lose optimism, increase time entering orders, and increase overall work time after implementation of an EHR [30]. As this was a study of perception about inefficient time, the concomitant EHR change could have undermined efficiency improvements from Squire. It is also possible that the EHR may have improved efficiency, confounding the results in the opposite direction; however, results from the interviews suggest that users attributed the noted efficiency gains to use of Squire.

Squire was custom developed and is currently available only in our institution. We anticipate that other institutions have similar challenges finding the most appropriate contact and therefore included substantial technical implementation details in the Methods section so other institutions could replicate Squire if desired.

Finally, we used a self-reported outcome of time spent searching for appropriate contacts that could be biased; a more direct measurement of this time may be more accurate. A future time-motion study could provide more robust measures of Squire’s impact on efficiency. Furthermore, in our regression model we collapsed the self-assessed outcome of time with unequal intervals into a mean time. Since these are in any case self-reported times, we do not believe this changes the results appreciably.

**Conclusions**

We developed a Web-based information retrieval app, Squire, and found that its use saved a modest amount of time per day searching for the correct contact in a hospital setting. While users also found the system highly usable, Squire did not improve the frustration in finding appropriate contacts, and the NPS was a mediocre 6.1. We also identified opportunities to iteratively improve Squire’s usability and features. While the study results were mixed, Squire has shown some value in improving the efficiency of finding the appropriate hospital care team member. As we iterate Squire based on the study findings, we have started extending Squire to other user groups and use cases. Squire may also be of interest to other institutions, so we described the technical design so that others can replicate Squire.

## Abbreviations

CSS: cascading style sheets
EHR: electronic health record
LDAP: lightweight directory access protocol
NPS: Net Promoter Score
REDCap: Research Electronic Data Capture
SOAP: simple-object access protocol
SSL: Secure Socket-Layer
SUS: System Usability Scale
